# Noninvasive Ultrasound Deep Brain Stimulation for the Treatment of Parkinson's Disease Model Mouse

**DOI:** 10.34133/2019/1748489

**Published:** 2019-07-09

**Authors:** Hui Zhou, Lili Niu, Long Meng, Zhengrong Lin, Junjie Zou, Xiangxiang Xia, Xiaowei Huang, Wei Zhou, Tianyuan Bian, Hairong Zheng

**Affiliations:** ^1^Paul C. Lauterbur Research Center for Biomedical Imaging, Institute of Biomedical and Health Engineering, Shenzhen Institutes of Advanced Technology, Chinese Academy of Sciences, China; ^2^Shenzhen College of Advanced Technology, University of Chinese Academy of Sciences, China

## Abstract

Modulating basal ganglia circuitry is of great significance in the improvement of motor function in Parkinson's disease (PD). Here, for the first time, we demonstrate that noninvasive ultrasound deep brain stimulation (UDBS) of the subthalamic nucleus (STN) or the globus pallidus (GP) improves motor behavior in a subacute mouse model of PD induced by 1-methyl-4-phenyl-1,2,3,6-tetrahydropyridine (MPTP). Immunohistochemical c-Fos protein expression confirms that there is a relatively high level of c-Fos expression in the STN-UDBS and GP-UDBS group compared with sham group (both p < 0.05). Furthermore, STN-UDBS or GP-UDBS significantly increases the latency to fall in the rotarod test on day 9 (p < 0.05) and decreases the time spent climbing down a vertical rod in the pole test on day 12 (p < 0.05). Moreover, our results reveal that STN-UDBS or GP-UDBS protects the dopamine (DA) neurons from MPTP neurotoxicity by downregulating Bax (p < 0.001), upregulating Bcl-2 (p < 0.01), blocking cytochrome c (Cyt C) release from mitochondria (p < 0.05), and reducing cleaved-caspase 3 activity (p < 0.01) in the ipsilateral substantia nigra (SN). Additionally, the safety of ultrasound stimulation is characterized by hematoxylin and eosin (HE) and Nissl staining; no hemorrhage or tissue damage is detected. These data demonstrate that UDBS enables modulation of STN or GP neural activity and leads to neuroprotection in PD mice, potentially serving as a noninvasive strategy for the clinical treatment of PD.

## 1. Introduction

Dysfunction in basal ganglia circuitry is largely responsible for the development of motor deficits in Parkinson's disease (PD) [1, 2]. The subthalamic nucleus (STN) and the internal segment of the globus pallidus (GPi) in the basal ganglia pathway have direct or indirect projections to the substantia nigra pars reticulata (SNpr), which ultimately influences motor function [3, 4]. Studies have shown that deep brain stimulation (DBS) of the STN or GPi ameliorates PD motor deficits, including akinesia, bradykinesia, rigidity, and tremor [5–11]. Basal ganglia DBS may improve cortical functioning by inhibiting excessive beta phase activation in the primary motor cortex of patients with PD [12]. Targeting the STN and GPi by DBS for the treatment of advanced PD has been approved by the Food and Drug Administration (FDA) [13]. Five-year follow-up studies have indicated that both STN-DBS [14] and GPi-DBS [15] result in long-term improvements in motor function and the quality of life in patients with PD. Randomized studies have also demonstrated that STN-DBS or GPi-DBS lead to similar improvements in motor function in advanced PD patients [16, 17]. However, DBS requires an invasive surgical procedure, which may lead to an increased risk of complications [18]. Noninvasive stimulation of the STN or GPi, therefore, is of critical importance for the treatment of PD.

Ultrasound is a mechanical wave [19], which can pass through an intact human skull and evoke neural activity [20–22]. Low intensity pulsed ultrasound (LIPUS) has shown great promise for the modulation of brain function and reversal of neurological and psychiatric dysfunction [23, 24]. LIPUS evokes motor response in mice when ultrasound was used to stimulate the motor cortex [25] and increases antisaccade latencies when ultrasound was delivered to the left frontal eye field in monkeys [26]. Ultrasound stimulation of the human somatosensory cortex has shown to enhance sensory discrimination [20]. Studies have also suggested that LIPUS may hold a great potential to be used as a new means of neurotherapeutics. LIPUS stimulation of olive-cerebellar pathways decreases tremor frequency in a rat model of essential tremor [27]. Seizure activity in the animal model of epilepsy is also suppressed by the LIPUS treatment [28–30] and depressive symptoms are reversed by LIPUS stimulation of the prelimbic cortex [31]. Furthermore, LIPUS stimulation of the ischemic cortex mitigates focal cerebral ischemia in a rat model of stroke induced by distal middle cerebral artery occlusion [24, 32]. Recently, we confirmed that LIPUS stimulation of the motor cortex increases the number of rearing in the open field test and reduces pole suspension time in the pole test in an acute mouse model of PD [33]. However, whether ultrasound stimulation of deep subcortical brain structures (STN or GPi) improves parkinsonian motor function has not been studied. Identifying the effects of STN-UDBS and GPi-UDBS on motor function and exploring basic, neuroprotective mechanisms for PD may promote the clinical applications of noninvasive ultrasonic neuromodulation.

**Figure 1:**
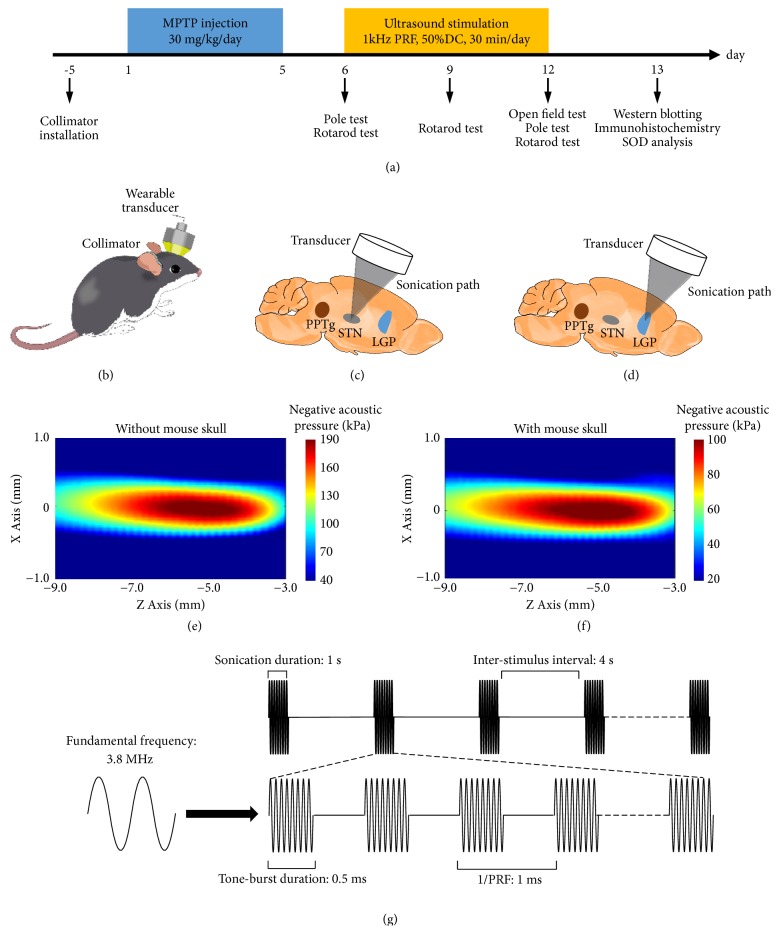
Experimental design and UDBS targets. (a) Timeline of the experiment; MPTP was administrated from day 1 to 5 and UDBS stimulation was delivered from day 6 to 12. Rotarod performance was assessed on day 6, 9, and 12. Pole test was performed on day 6 and 12, and the open field test was conducted on day 12. (b) The wearable ultrasound for deep brain stimulation of the STN (c) or GP (d). Acoustic intensity distributions in the longitudinal plane without mouse skull (e) and with mouse skull (f). (g) Schematic of UDBS parameters with 1 kHz pulse repetition frequency (PRF) and 50% duty cycle (DC).

In the present study, we investigated the treatment effects of STN-UDBS or GP-UDBS on MPTP-induced motor impairments in a mouse model of PD and explored a possible mechanism for these effects. Here, we demonstrated that noninvasive UDBS of STN or GP is capable of enhancing motor function in a subacute PD mouse model.  Figure 1(a) shows the timeline of the experiment. We first built a subacute PD mouse model induced by MPTP, which causes a reliable lesion of the nigrostriatal dopaminergic pathway and recapitulates the pathological features of PD [34]. We then fabricated a wearable single-element ultrasound transducer, which had a millimeter-scale focus. Transcranial ultrasound (3.8 MHz fundamental frequency, 50% duty cycle, 1 kHz pulse repetition frequency (PRF), 0.5 ms tone burst duration (TBD), 1 s sonication duration (SD), 4 s inter-stimulation interval (ISI), 30 min per day, and total 7 days) was applied to the STN or GP in awake, freely moving mice (Figures 1(b), 1(c), 1(d) and 1(g)). Lastly, behavioral tests, antioxidative detection, immunohistochemistry, and western blotting were carried out to evaluate the effects of STN-UDBS or GP-UDBS in MPTP mouse model of PD. Our results revealed that LIPUS stimulation of the STN or GP recovered rotarod performance in the rotarod test and improved locomotor activity in the pole test in MPTP-treated mice. Moreover, UDBS attenuated cell apoptosis by promoting an increased ratio of Bcl-2/Bax, which further inhibited Cyt C release from mitochondria and downregulated cleaved-caspase 3 activity.

## 2. Results

### 2.1. UDBS Increases c-Fos Expression in the STN and GP of Mice

**Figure 2:**
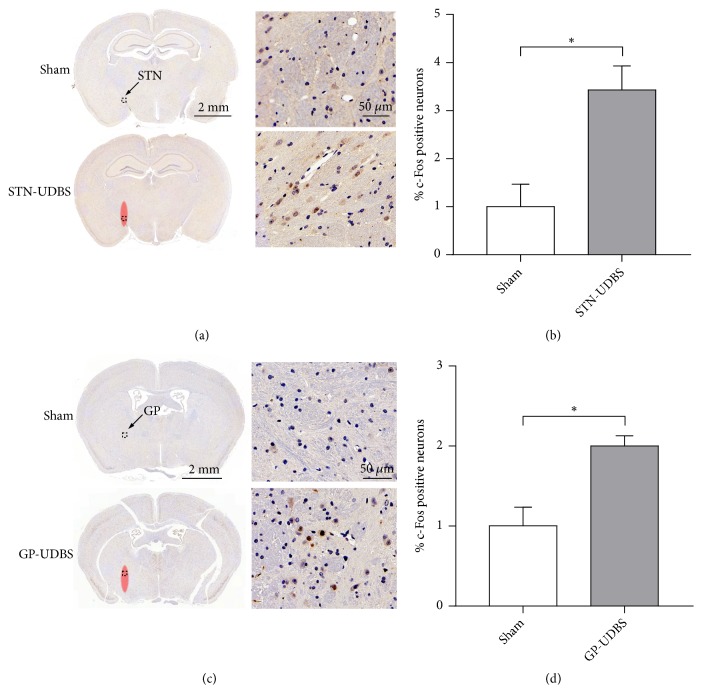
UDBS increases c-Fos positive neurons in the STN and the GP. Respective c-Fos staining in the STN (a) and the GP (c). Cells with nuclear c-Fos staining (brown cell nuclei) represent cells that respond to ultrasound stimulation, and UDBS targeted regions are indicated by red ellipses. The percent of c-Fos positive neurons in the STN (b) and the GP (d) is normalized with sham group (independent sample t-test, *∗*p < 0.05, mean ± SEM, n = 3 per group).

Neural activity in the STN-UDBS or GP-UDBS group is quantitatively assessed by immunohistochemistry staining of c-Fos, which is widely used as a marker of neuronal activity [35].  Figure 2 shows the c-Fos expression in the STN and GP after ultrasound stimulation. Figures 2(a) and 2(b) indicate that the expression of c-Fos positive neurons in the STN after 30 min of STN-UDBS is significantly increased compared with sham group (p = 0.023). Similarly, a significant increment of c-Fos positive neurons in the GP is observed in GP-UDBS group compared with sham group (p = 0.02), as shown in Figures 2(c) and 2(d). These results indicate that UDBS effectively activates neurons in the STN and GP. The c-Fos expression in the route of ultrasound stimulation is shown in Supplementary [Supplementary-material supplementary-material-1].

### 2.2. Effect of UDBS on Motor Performance

#### 2.2.1. Rotarod Test

**Figure 3:**
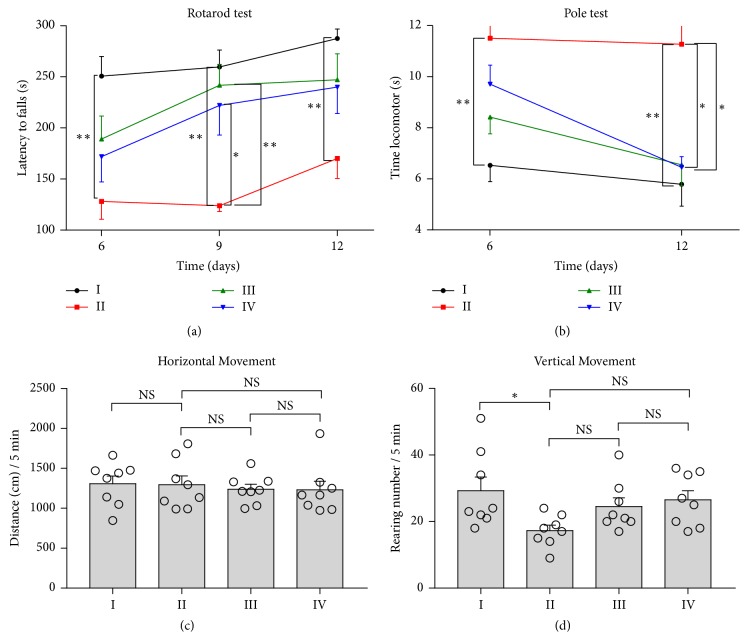
UDBS of STN or GP improves motor behavior in MPTP mice. (a) UDBS improves motor performance in the rotarod test. The latency to fall is significantly decreased in group II compared with that in group I. The time on the rod is increased in groups III and IV compared with that in group II on day 9. There is no significant difference between groups III and IV on day 6, 9, and 12. (b) UDBS recovers the time to climb down in the pole test. The time spent climbing down the pole is increased in group II compared with that in group I on day 6 and 12. The time spent climbing down is significantly decreased in groups III and IV compared with that in group II on day 12. (c) No differences in horizontal movement are found among all groups. (d) UDBS improves vertical movement on day 12. The rearing number is significantly decreased in group II compared with that in group I on day 12 and no significantly increased in groups III and IV compared with group II. (group I: control-sham, group II: MPTP-sham, group III: MPTP-STN-UDBS and group IV: MPTP-GP-UDBS; one–way ANOVA with Tukey's post hoc: *∗*p < 0.05, *∗∗*p < 0.01, *∗∗∗*p < 0.001; Kruskal Wallis nonparametric ANOVA for the rotarod test on day 9 and 12; mean ± SEM, n = 9 per group in (a) and (b), n = 8 per group in (c) and (d)).

Mice are randomly divided into the following groups: (I) control-sham, (II) MPTP-sham, (III) MPTP-STN-UDBS, and (IV) MPTP-GP-UDBS, and the time points for behavioral tests are shown in Figure 1(a). In this study, the rotarod test is performed to assess the motor coordination in PD model mice, and the latency to fall from the rod is recorded to evaluate the degree of impairment.  Figure 3(a) shows that the latency to fall is significantly decreased in group II as compared with group I on day 6 (group I: 250.78 ± 19.10 s; group II: 128.17 ± 17.48 s, p = 0.001), day 9 (group I: 259.72 ± 16.62 s; group II: 123.83 ± 5.71 s, p = 0.001), and day 12 (group I: 287.56 ± 9.30 s; group II: 170.11 ± 19.59 s, p = 0.003). Groups III and IV demonstrate improved to fall from the rod compared with group II on day 6 (group III: 189.22 ± 22.35 s, p = 0.193; group IV: 171.83 ± 24.69 s, p = 0.470); these times further improve on day 9 (group III: 241.72 ± 20.54 s, p = 0.009; group IV: 222.11 ± 29.07 s, p = 0.042), and day 12 (group III: 247.11 ± 25.41 s, p = 0.119; group IV: 239.94 ± 25.79 s, p = 0.250). There are no significant difference in the latency to fall between groups III and IV on day 6 (p = 0.936), day 9 (p = 1.000), and day 12 (p = 1.000) (Supplementary Movies [Supplementary-material supplementary-material-1], [Supplementary-material supplementary-material-1] and [Supplementary-material supplementary-material-1]).

#### 2.2.2. Pole Test

We adopt the pole test to assess the effects of UDBS on the motor balance of PD model mice. The results of the pole test on day 6 and 12 are depicted in Figure 3(b) and Supplementary [Supplementary-material supplementary-material-1]. The time mice spent climbing down the pole is significantly increased in group II compared with group I on day 6 (group I: 6.53 ± 0.63 s; group II: 11.51 ± 1.12 s, p = 0.001) and day 12 (group I: 5.79 ± 0.85 s; group II: 11.27 ± 1.88 s, p = 0.009). Mice in groups III and IV spend less time climbing down the pole than did mice in group II on day 6 (group III: 8.43 ± 0.66 s, p = 0.053; group IV: 9.71 s ± 0.74 s, p = 0.417) and day 12 (group III: 6.55 ± 0.82 s, p = 0.029; group IV: 6.45 s ± 0.42 s, p = 0.025). There are no significant difference in the time spend climbing down the pole between groups III and IV on day 6 (p = 0.679) and day 12 (p = 1.000).

#### 2.2.3. Open Field Test

Neither MPTP nor ultrasound stimulation alters the horizontal movement in the open field test (OFT), as shown in Figure 3(c). The rearing number is significantly decreased in group II compared with group I (group I: 29.25 ± 4.11; group II: 16.88 ± 1.65, p = 0.032). The rearing number increases in groups III and IV compared with that in group II (group III: 23.63 ± 2.68, p = 0.397, and group IV 26.13 s ± 2.99, p = 0.152). There are no significant difference in rearing number between groups III and IV on day 12 (p = 0.934), as shown in Figure 3(d).

### 2.3. Neuroprotective Effect of UDBS on Nigrostriatal Degeneration

**Figure 4:**
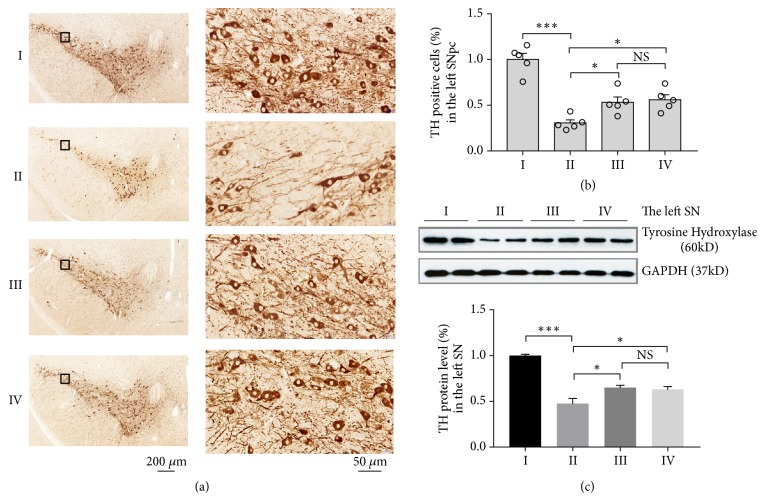
Neuroprotective effects of STN-UDBS or GP-UDBS. (a) Immunohistochemistrical staining for TH positive neurons in the left SNpc. (b) The number of TH positive neurons in the left SNpc is significantly decreased in group II compared with group I and significantly increased in groups III and IV compared with group II. (c) Western blot analysis of TH protein level in the left SN. TH protein level decreases after MPTP injection and UDBS increases TH protein level in group III and IV compared with group II (group I: control-sham, group II: MPTP-sham, group III: MPTP-STN-UDBS and group IV: MPTP-GP-UDBS; one–way ANOVA with Tukey's post hoc: *∗*p < 0.05, *∗∗*p < 0.01, *∗∗∗*p < 0.001, mean ± SEM, n = 5 per group in (a), n = 4 per group in (c)).

To investigate the neuroprotective effects of UDBS on the nigrostriatal pathway, we performed tyrosine hydroxylase (TH) immunohistochemistry and western blot analysis for the section of the SN and striatum, respectively. Figures 4(a) depicts the immunohistochemical TH staining in the left substantia nigra pars compacta (SNpc). The number of TH positive neurons in group II is significantly decreased as compared with group I (group I: 1.00 ± 0.07; group II: 0.31 ± 0.03, p < 0.001). The number of TH positive neurons is significantly increased in groups III and IV as compared with group II (group III: 0.53 ± 0.06, p = 0.046; group IV: 0.56 ± 0.05, p = 0.023). No significant difference is detected in the number of TH positive neurons between groups III and IV (p = 0.985). The impact of UDBS on striatal TH neuritis in Supplementary [Supplementary-material supplementary-material-1].

We further evaluate TH protein level in the left SN. As shown in Figure 4(c), TH protein level in group II is significantly decreased as compared with group I (group I: 1.00 ± 0.01; group II: 0.47 ± 0.06, p < 0.001). TH protein level is increased in groups III and IV compared with group II (group III: 0.65 ± 0.02, p = 0.018; group IV: 0.63 ± 0.03, p = 0.038). There is no significant difference in TH protein level between groups III and IV (p = 0.973). The impact of UDBS on TH protein level in the right SN and striatum is shown in Supplementary Figures [Supplementary-material supplementary-material-1], and [Supplementary-material supplementary-material-1].

### 2.4. UDBS Suppresses Cell Apoptosis Induced by MPTP

**Figure 5:**
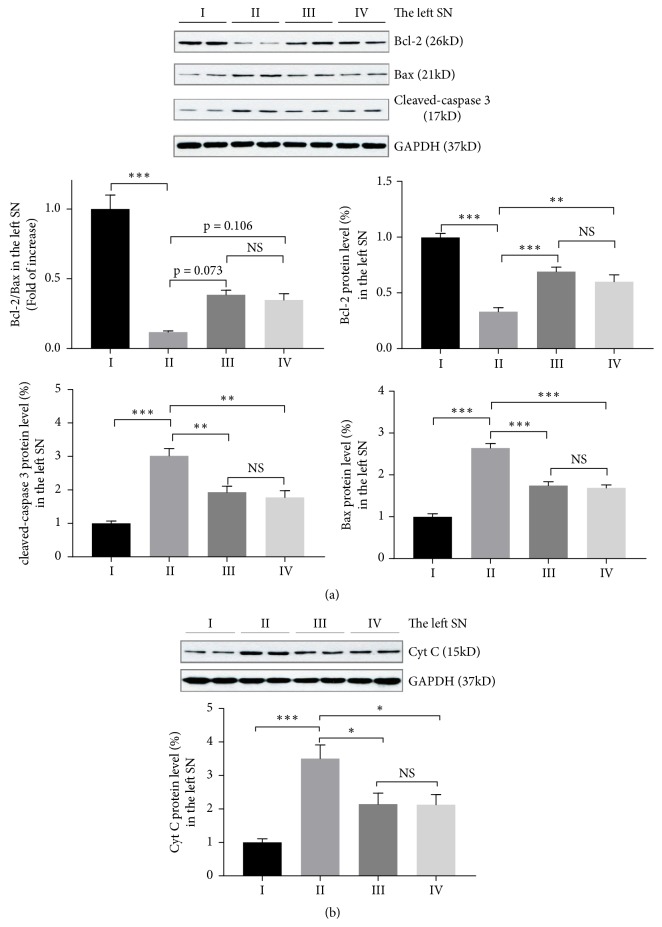
STN-UDBS or GP-UDBS suppresses MPTP induced cell apoptosis. (a) Bcl-2 is downregulated and Bax is upregulated after MPTP injections. These levels are restored by ultrasound stimulation. Besides, UDBS reverses cleaved-caspase 3 activity induced by MPTP treatment. (b) Cyt C release from mitochondria increases after MPTP treatment, and decreases following ultrasound stimulation. (group I: control-sham, group II: MPTP-sham, group III: MPTP-STN-UDBS and group IV: MPTP-GP-UDBS; one–way ANOVA with Tukey's post hoc: *∗*p < 0.05, *∗∗*p < 0.01, *∗∗∗*p < 0.001 for Bcl-2, Bax, Cyt C and cleaved-caspase 3 analysis; Kruskal Wallis nonparametric ANOVA for Bcl-2/Bax analysis; mean ± SEM, n = 8 per group for Bcl-2 and Bax, n = 4 per group for cleaved-caspase 3 and Cyt C).

MPTP promotes DA neuron loss in MPTP mice by inhibiting multienzyme complex I within the mitochondria, which further induce cell apoptosis in the SNpc [36]. Mitochondria-mediated apoptosis is associated with the balance of Bcl-2 and Bax [37, 38]. Bcl-2 locates at the mitochondrial membranes and is capable of inhibiting neuronal death through blocking Cyt C release from mitochondria [39]. Conversely, Bax regulates Cyt C release from the mitochondria and promotes cell apoptosis [40]. Cyt C release from the mitochondria activates caspase 3 and leads to cell apoptosis [41]. In the present study, we observe that Bcl-2 decrease and Bax increase after MPTP treatment (Bcl-2: group I, 1.00 ± 0.04; group II: 0.33 ± 0.04, p < 0.001; Bax: group I, 1.00 ± 0.07; group II: 2.64 ± 0.11, p < 0.001; Bcl-2/Bax: group I, 1.00 ± 0.10; group II: 0.12 ± 0.01, p < 0.001). Meanwhile STN-UDBS or GP-UDBS upregulates Bcl-2 and downregulates Bax in groups III and IV compared with group II (Bcl-2: group III: 0.69 ± 0.04, p < 0.001; group IV: 0.60 ± 0.06, p = 0.001; Bax: group III: 1.74 ± 0.10, p < 0.001; group IV: 1.69 ± 0.07, p < 0.001; Bcl2/Bax: group III: 0.39 ± 0.03, p = 0.073; group IV: 0.35 ± 0.05, p = 0.106, Figure 5(a)). There is no significant difference in the levels of Bcl-2 and Bax between groups III and IV (p = 0.509 for Bcl-2; p = 0.970 for Bax; and p = 1.000 for Bcl-2/Bax).

We also measure Cyt C and cleaved-caspase 3 levels and found that protein levels of both Cyt C and cleaved-caspase 3 are significantly increased following the administration of MPTP in group II compared with group I (Cyt C: group I, 1.00 ± 0.11; group II: 3.51 ± 0.41, p < 0.001; cleaved-caspase 3: group I, 1.00 ± 0.08; group II: 3.02 ± 0.22, p < 0.001). However, these increments are inhibited by STN-UDBS and GP-UDBS (Cyt C: group III: 2.14 ± 0.33, p = 0.039; group IV: 2.13 ± 0.31, p = 0.037, Figure 5(b); cleaved-caspase 3: group III: 1.93 ± 0.18, p = 0.005; group IV: 1.78 ± 0.20, p = 0.002, Figure 5(a)). There are no significant differences in the levels of Cyt C and cleaved-caspase 3 between group III and IV (p = 1.000 for Cyt C and p = 0.925 for cleaved-caspase 3). The impact of UDBS on mitochondrial dysfunction and apoptosis in the right SN and striatum are shown in Supplementary Figures [Supplementary-material supplementary-material-1], [Supplementary-material supplementary-material-1] and [Supplementary-material supplementary-material-1], respectively.

### 2.5. UDBS Safety Assessment

**Figure 6:**
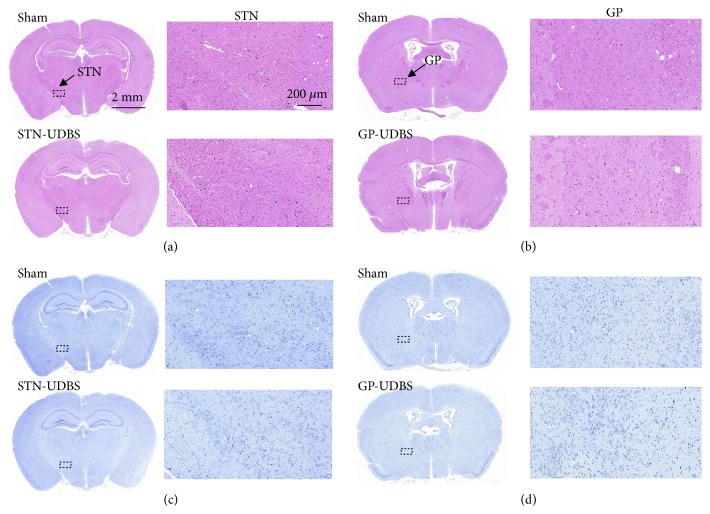
Histological evaluation in STN and GP after ultrasound stimulation. Representative HE staining (a) and (b), Nissl staining (c) and (d) indicated no tissue damage caused by ultrasound stimulation.

Hematoxylin and eosin (HE) staining is widely used to assess the presence of hemorrhaging or tissue damage and Nissl staining to visualize neurons. In this study, HE and Nissl staining were performed to evaluate the safety of UDBS. Figures 6(a) and 6(b) depict representative HE staining images and no hemorrhaging or tissue damage is observed in the STN-UDBS or GP-UDBS group after seven days of stimulation. Figures 6(c) and 6(d) show that neuronal density appeared to be normal throughout the brain in all groups. Therefore, the UDBS parameter used in this study is safe for the treatment of PD mice.

## 3. Discussion

Our results demonstrate that STN-UDBS and GP-UDBS improve motor function in MPTP mouse model of PD and protect TH positive neurons in the SNpc against MPTP-induced cell death. The results reveal that UDBS has neuroprotective effects against MPTP neurotoxin by promoting the ratio of Bcl-2/Bax and inhibiting Cyt C release from mitochondria, thereby suppressing cell apoptosis. UDBS not only allows for noninvasive brain modulation, but also may serve as a powerful tool for the noninvasive treatment of PD.

Although the MPTP model does not completely mimic all pathological features of PD, it does recapitulate basal ganglia dysfunction and thus serves as a suitable tool for the assessment of STN-UDBS and GP-UDBS efficacy in treating PD model mouse. MPTP subacute treatment causes dopaminergic lesion and deplete striatal dopamine in C57BL mice within 21 days after MPTP administration [42]. Consistent with the previous study, 5 days of MPTP administration cause degeneration of DA neurons in the SNpc by day 12 (Figures 4(a) and 4(b)). In this study, MPTP-treated mice spent less time on the rod in the rotarod test and took longer time to descend the pole in the pole test, reflecting impairments in motor coordination and movement balance due to MPTP. The parkinsonian motor symptoms in these mice were also consistent with their reduced TH protein levels (Figures 4(c) and 4(d); Supplementary [Supplementary-material supplementary-material-1]). On day 12, both STN-UDBS and GP-UDBS promoted behavioral recovery in the rotarod test and pole test (Figures 3(a) and 3(b)). Besides, we found that MPTP mice had better performance in the pole test at 6 hours after UDBS stimulation, as shown in Supplementary [Supplementary-material supplementary-material-1]. We chose the primary visual cortex (V1) as a control target and found that ultrasound stimulation of V1 has little impact on the time to climb down the pole in the pole test as shown in Supplementary [Supplementary-material supplementary-material-1]. The results suggested that modulation of non-STN related regions are not helpful for movement performance. Additionally, both STN-UDBS and GP-UDBS mitigated DA neurons loss in the SNpc (Figures 4(a) and 4(b), Supplementary [Supplementary-material supplementary-material-1]). These results revealed the neuroprotective effects of STN-UDBS and GP-UDBS on the nigrostriatal DA pathways.

The mechanism underlying ultrasonic neuromodulation effects in PD remains unclear. MPTP simulates mitochondrial apoptotic pathways and ultimately induces dopaminergic cell death in the SN [43, 44]. Evidence indicates that Bcl-2 and Bax are involved in mitochondria-mediated apoptosis in neurodegenerative diseases [37, 41]. Bcl-2 has neuroprotective effects against the depletion of striatal dopamine in the context of the MPTP neurotoxin [45]. Bax ablation attenuates DA neuron apoptosis induced by MPTP damage [46]. In a MPTP mouse model, proapoptotic protein Bax is upregulated, whereas the antiapoptotic protein Bcl-2 is downregulated. Thus, the permeability of the mitochondrial membrane may thus be damaged by the decreased ratio of Bcl-2/Bax, which leads to Cyt C release from the mitochondria and activation of caspase 3 [41]. Activation of caspase 3 ultimately results in the death of DA neurons in PD [47]. Therefore, a treatment that alleviates apoptosis may prevent DA neurons loss in MPTP-treated mice. Besides, SOD involves in the conversion of superoxide in cytoplasm and mitochondria and provides the neuroprotective effect for DA neurons in the SNpc [48]. Our previous work has confirmed that ultrasonic stimulation of the motor cortex enhanced striatal SOD content. More importantly,* in vitro* assessments have verified that LIPUS promotes Bcl-2/Bax ratios and prevents Cyt C release from mitochondria; this further suppresses cleaved-caspase 3 activity [49], which is linked to apoptosis and neuronal death. In the present study, we found that stimulation of the STN or GP inhibited Cyt C release and cleaved-caspase 3 activity and increased the ratio of Bcl-2/Bax in the SN and striatum (Figure 5; Supplementary Figures [Supplementary-material supplementary-material-1], [Supplementary-material supplementary-material-1] and [Supplementary-material supplementary-material-1]). Additionally, there is no significant difference in striatal SOD between STN-UDBS and GP-UDBS (Supplementary [Supplementary-material supplementary-material-1]). We would like to increase the sample size to verify these results. Other studies have similarly shown that LIPUS stimulation of the rat thalamus increased serotonin and dopamine in the frontal lobe [50]. LIPUS may also increase brain derived neurotrophic factor (BDNF) levels, which is critical for neuronal survival and plasticity in the murine hippocampus [51]. Further study of the mechanism underlying the effects of ultrasound stimulation in PD is required.

Although STN-DBS is widely used for the clinical treatment of PD, randomized controlled studies have suggested that motor deficit treatment outcomes do not significantly differ between STN-DBS and GPi-DBS [52]. A large number of* in vivo* studies have also illustrated that either STN-DBS or GPi-DBS is able to improve motor performance in rats administrated with 6-OHDA [53, 54]. In the present study, we found that STN-UDBS and GP-UDBS (3.8 MHz fundamental frequency, 50% duty cycle, 1 kHz pulse repetition frequency, 0.5 ms tone burst duration, 1 s sonication duration, and 4 s inter stimulation interval) ameliorated motor deficits in the subacute MPTP mice. Furthermore, we did not identify any significant differences in the results from behavioral tests or western blot analyses between STN-UDBS and GP-UDBS animals in the current study. To further verify the effects of STN-UDBS and GP-UDBS in PD, other PD animal models, such as the 6-OHDA model, need to be included in the future studies. Previous studies have indicated that ultrasound wave with 1 kHz PRF and 50% DC is able to elicit motor response in rat [25] and rabbit [55]. Further studies are needed to explore the impact of different ultrasound parameters on the ultrasound neuromodulation effects.

The three main effects induced by ultrasound are cavitation, thermal, and mechanical effect. In our study, the negative peak pressure (0.1 MPa) caused by ultrasound stimulation was far below the threshold for the occurrence of cavitation in the absence of microbubbles (40 MPa) [56], suggesting that the cavitation effect was not likely to be involved in this study. Additionally, the temperature elevation induced by ultrasound in our study was less than 0.2°C. Previous studies indicated that a 0.75°C rise in temperature induced by ultrasound stimulation has little impact on the biological effects in hippocampus tissue [57]. Our previous studies have shown that the mechanical effect, other than the thermal effect, played an important role in the neuromodulation for* Caenorhabditis elegans* [58],* ex vivo *brain slices [59, 60], and rodents [61, 62]. Lastly, according to HE and Nissl staining assessments, there was no tissue damage along the acoustic beam path after 7 days of ultrasound stimulation (Figure 6). Both mechanical index (MI, 0.17) and Ispta (180 mW/cm^2^) were far below current FDA clinical ultrasound imaging thresholds (MI = 1.9 and Ispta = 720 mW/cm^2^), ensuring the safety of ultrasound stimulation used here [63].

In conclusion, our results demonstrate that STN-UDBS and GP-UDBS improved motor functioning and protected DA neurons in MPTP mice through antiapoptotic effects. No significant differences in treatment effects were found between the stimulation of the two targets (STN and GP). Our findings indicate that either STN or GP may serve as ideal targets for noninvasive ultrasound deep brain stimulation for the treatment of PD.

## 4. Materials and Methods

### 4.1. Animal Preparation

All animal protocols described in this work (Certificate number: SIAT-IRB-150213-YGS-ZHR-A0094-2) were approved by the Institutional Ethical Committee of Animals Experimentation of Shenzhen Institutes of Advanced Technology (Chinese Academy of Sciences). A total of 120 male C57BL/6J mice (8 weeks old, Beijing Vital River Laboratory Animal Technology Co., Ltd.) were used in the experiment. Mice received intraperitoneal (i.p.) injections of MPTP (30 mg/kg body weight, once daily; Sigma-Aldrich) or an equivalent volume of saline from day 1 to day 5 [34] as shown in Figure 1(a). A collimator was fixed onto the mouse skull as previously described [33], and used to guide ultrasound focus to the STN (coordinates from bregma: -2.06 mm anterior/ posterior, -1.50 mm medial/lateral, and -4.50 mm dorsal/ventral) or GP (coordinates from bregma: -0.34 mm anterior/posterior, -1.80 mm medial/lateral, and -4.00 mm dorsal/ventral) (Figures 1(b), 1(c), and 1(d)). Ultrasound stimulation was conducted from day 6 to 12 in groups III and IV. Groups I and II included sham controls in which animals wore an ultrasound transducer while the power was turn off. Behavioral tests were conducted on days 6, 9, and 12. All mice were sacrificed on day 13 and tissues were used for western blot analysis, TH immunohistochemistry, and striatal total superoxide dismutase (SOD) content detection.

The UDBS system and transducer characteristics were summarized in Supplementary [Supplementary-material supplementary-material-1]. The ultrasound parameters (Figure 1(g)) used here were as sollows: 3.8 MHz fundamental frequency, 1 kHz PRF, 50% DC, 1 s SD, and 4 s ISI for 30 min daily (Figure 1(g)). The acoustic intensity maps in the longitudinal plane (Figures 1(e) and 1(f)) and in the transversal plane (Supplementary [Supplementary-material supplementary-material-1]) were measured via a 3D ultrasound intensity measurement system as previously described [33]. The acoustic intensity distribution had a 0.8 × 3 mm full width at half maximum (FWHM) focal spot. The negative acoustic pressure was 0.19 MPa with a spatial-peak temporal-average intensity (I_spta_) of 430 mW/cm^2^ in free space. After passing through the mouse skull, the negative peak acoustic pressure was 0.10 MPa, and the I_spta_ was 180 mW/cm^2^. The attenuation of acoustic pressure through the mouse skull was 61%. The full width at half-maximum was 0.8 mm.

### 4.2. Behavioral Tests

#### 4.2.1. Rotarod Test

A rotarod apparatus (YLS-4C Zhenghua, Anhui, China) was used to assess motor coordination and balance in mice. Before MPTP injection, all mice were trained on the rotarod to reach stable latency to fall. The rotating rod was set to automatically stop at 300 s. On the test day, each mouse was placed on the rod rotating at a speed of 40 rotations per minute (rpm). The latency to fall from the rod was automatically recorded by the apparatus when the mouse fell from the rod to land at its base. Each mouse was tested twice with a 30 min rest period. Each animal's average latency to fall was then calculated for further analyses. The rotarod test was performed on days 6, 9, and 12 (Figure 1(a)), respectively.

#### 4.2.2. Pole Test

The pole test was used to evaluate the movement performance of PD mice on days 6 and 12 (Figure 1(a)). Briefly, the time that mice spent climbing from the top of 50 cm tall and 1 cm diameter pole to its base was recorded. Mice were pretrained on day 5. Each mouse was then tested twice and the average time was calculated for further analyses.

#### 4.2.3. Open Field Test

Locomotor behavior was assessed using the open field test on day 12 (Figure 1(a)). In detail, mice were placed at the center of a PMMA square arena in a quiet room (30 cm × 30 cm × 30 cm) and allowed to habituate for 10 min. Subsequently, the total distance traveled in a 5 min period was recorded as spontaneous locomotor activity using Smart 3.0 software (Panlab, Spain, Barcelona). The rearing behavior was assessed manually. The arena was then cleaned with 75% alcohol between trials.

### 4.3. Immunohistochemistry

C-Fos immunohistochemistry was performed as previously described [35]. To confirm the presence of TH positive neurons in the SN, brain tissues from the four groups (n = 5 for each group) were collected on day 13. Brain slices were incubated in rabbit anti-TH antibody (1:750, Abcam, ab112) overnight at 4°C and then incubated in goat anti-rabbit HRP IgG (G23303, Wuhan Servicebio Technology Co., Ltd., China) for 30 min at 37°C. Quantitative analysis of the total number of TH positive neurons in the SNpc was conducted. All images were viewed and captured with a digital camera (ds-U3, Nikon Instruments Inc., Tokyo, Japan).

### 4.4. Western Blot Analysis

The SN and striatum were prepared in RIPA buffer containing phosphate and protease inhibitor cocktails. After 30 min in an ice-bath, tissue lysate was obtained by centrifugation at 12,000 rpm for 10 min. The tissue lysate was mixed in a loading buffer and boiled for 5 min. Then, equivalent amounts of protein were separated on a 10% SDS-polyacrylamide gel and transferred onto PVDF membranes. Membranes were blocked with 5% BSA in Tris-buffered saline. Anti-TH (1:300, Abcam, ab112), anti-Bcl2 (1:100, Santa, Sc492), anti-Bax (1:500, Abcam, ab32503), Cyt C (1:500, Abcam, ab13575), and cleaved-caspase 3 (1:500, CST, 9662) were diluted in 1% BSA and incubated overnight at 4°C, followed by incubation for 2 h at room temperature with anti-rabbit IgG HRP (1:5000, Abcam, ab6721) or anti-mouse IgG HRP (1:5000, Abcam, ab6789). Immunoblotting was stripped and reprobed with antibodies to GAPDH (ab181602, 1:3000) as an internal control. Blots were quantified by an Epson V330 Photo Scanner (Seiko Epson Co., Nagano, Japan) and densitometry was performed with Quantity One v.4.6.2.

### 4.5. Assessment of T-SOD in the Striatum

The striatum in each of the four groups (n = 8 per group) were homogenized in cold saline and SOD activity was detected using test kits (catalog no. A001-2-1, Nanjing Jiancheng Bioengineering Institute, Nanjing, China) according to manufacturer instruction.

### 4.6. Safety and Temperature Evaluation

Standard HE and Nissl staining procedures were performed for mice in all groups [64]. In detail, a total of nine healthy mice (n = 3 per group) were prepared for histological assessment. Each brain was embedded in paraffin and brain slices (4 *μ*m) encompassing the STN in the STN-UDBS group and the GP in the GP-UDBS group were sectioned with a pathologic microtome (Leica, RM2016). To assess heat accumulation in the mouse skull induced by UDBS, we measured the temperature of an* in vitro *mouse skull after UDBS stimulation using an infrared thermal imager (R300, NEC Avio, Tokyo, Japan).

### 4.7. Statistical Analyses

All experimental data were expressed as mean ± standard error of mean (SEM). All analyses were performed via independent sample t-test, one-way analyses of variance (ANOVAs) with Tukey's or Bonferroni's post hoc test for parametric analysis, and Kruskal Wallis for nonparametric analysis (SPSS statistics 22). A value of p < 0.05 was considered statistically significant.

## Data Availability

All data needed to support the conclusions of this work are available within paper and Supplementary Materials.

## Supplementary Materials

Supplementary Materials Supplementary Figure 1: schematic of the experiment apparatus and the structure of a wearable transducer. Supplementary Figure 2: neuroprotective effects of STN-UDBS or GP-UDBS in MPTP mice. Supplementary Figure 3: UDBS suppresses MPTP-induced apoptosis in right SN. Supplementary Figure 4: TH expression in left striatum. Supplementary Figure 5: STN-UDBS or GP-UDBS suppresses MPTP-induced apoptosis in left striatum. Supplementary Figure 6: UDBS has neuroprotective effects in right striatum. Supplementary Figure 7: UDBS increases T-SOD in left striatum. Supplementary Figure 8: C-Fos expression in the route of ultrasound stimulation. Supplementary Figure 9: the effect of STN-UDBS on motor function in the pole test at 6 hours after ultrasound stimulation. Supplementary Figure 10: TUNEL staining in STN and GP after ultrasound stimulation indicated that no apoptosis is induced by ultrasound stimulation. Supplementary Movie 1: MPTP-sham mice performance in the rotarod test on day 9. Supplementary Movie 2: the effect of STN-UDBS on behavior performance in the rotarod test on day 9. Supplementary Movie 3: the effect of GP-UDBS on behavior performance in the rotarod test on day 9. Supplementary Movie 4: The effect of STN-UDBS or GP-UDBS on behavior performance in the pole test on day 12.
