# Supplementary material for: Noninvasive Ultrasound Deep Brain Stimulation for the Treatment of Parkinson's Disease Model Mouse
Source: Research (Wash D C). 2019 Jul 9;2019:1748489. doi: 10.34133/2019/1748489 (PMC6750068; doi:10.34133/2019/1748489)
Supplement: Supplementary Materials — Supplementary Figure 1: schematic of the experiment apparatus and the structure of a wearable transducer. Supplementary Figure 2: neuroprotective effects of STN-UDBS or GP-UDBS in MPTP mice. Supplementary Figure 3: UDBS suppresses MPTP-induced apoptosis in right SN. Supplementary Figure 4: TH expression in left striatum. Supplementary Figure 5: STN-UDBS or GP-UDBS suppresses MPTP-induced apoptosis in left striatum. Supplementary Figure 6: UDBS has neuroprotective effects in right striatum. Supplementary Figure 7: UDBS increases T-SOD in left striatum. Supplementary Figure 8: C-Fos expression in the route of ultrasound stimulation. Supplementary Figure 9: the effect of STN-UDBS on motor function in the pole test at 6 hours after ultrasound stimulation. Supplementary Figure 10: TUNEL staining in STN and GP after ultrasound stimulation indicated that no apoptosis is induced by ultrasound stimulation. Supplementary Movie 1: MPTP-sham mice performance in the rotarod test on day 9. Supplementary Movie 2: the effect of STN-UDBS on behavior performance in the rotarod test on day 9. Supplementary Movie 3: the effect of GP-UDBS on behavior performance in the rotarod test on day 9. Supplementary Movie 4: The effect of STN-UDBS or GP-UDBS on behavior performance in the pole test on day 12. [file 1748489.f1.zip › 1748489.f1/Zhou_supplyment-file.docx]

Supplementary Materials for *Research*

**Noninvasive Ultrasound Deep Brain Stimulation for the Treatment of Parkinson's Disease Model Mouse**

Hui Zhou,^1,2,#^, Lili Niu,^1,#^, Long Meng,^1,#^, Zhengrong Lin,^1^, Junjie Zou,^1^, Xiangxiang Xia^1^, Xiaowei Huang^1^, Wei Zhou^1,2^, Tianyuan Bian^1^, Hairong Zheng^1,*^

^1^ Paul C. Lauterbur Research Center for Biomedical Imaging, Institute of Biomedical and Health Engineering, Shenzhen Institutes of Advanced Technology, Chinese Academy of Sciences

^2^ Shenzhen College of Advanced Technology, University of Chinese Academy of Sciences’

^#^These authors contributed equally to this work and are co-first authors

***Author for Correspondence:**

Hairong Zheng, Ph.D.

Professor, Paul C. Lauterbur Research Center for Biomedical Imaging, Chinese Academy of Sciences

1068 Xueyuan Ave., Nanshan District, Shenzhen, China, 518055

Email: [hr.zheng@siat.ac.cn](mailto:hr.zheng@siat.ac.cn) Tel: +86-755-86585278 Fax: 86-755-86392299


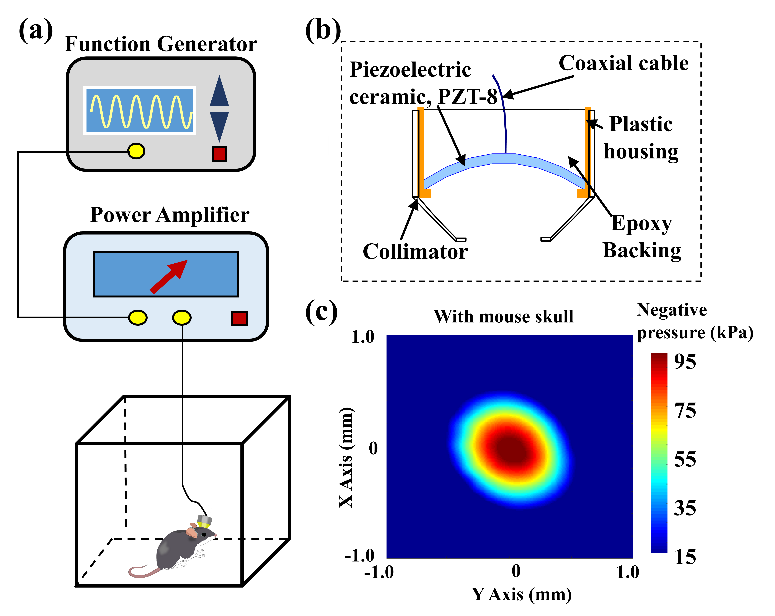


**Supplementary Figure 1** Schematic of the experiment apparatus and the structure of a wearable transducer. (a) UDBS system consisted of a two-channel generator and a 100 W radio frequency amplifier. (b) The wearable transducer consisted of a concave piezoelectric ceramic (Siansonic, Beijing, China), a plastic housing, epoxy backing, and a coaxial cable. The size of the piezoelectric ceramic was 12 mm in diameter, 0.5 mm in thick, and a radius-of-curvature of 12 mm. The structural parameters of the collimator were as follows: upper inner diameter of 13 mm, bottom inner diameter of 4.4 mm, total height of 12 mm, and distance from ultrasound to bottom 4 mm. (c) Acoustic intensity distribution in the transversal plane.


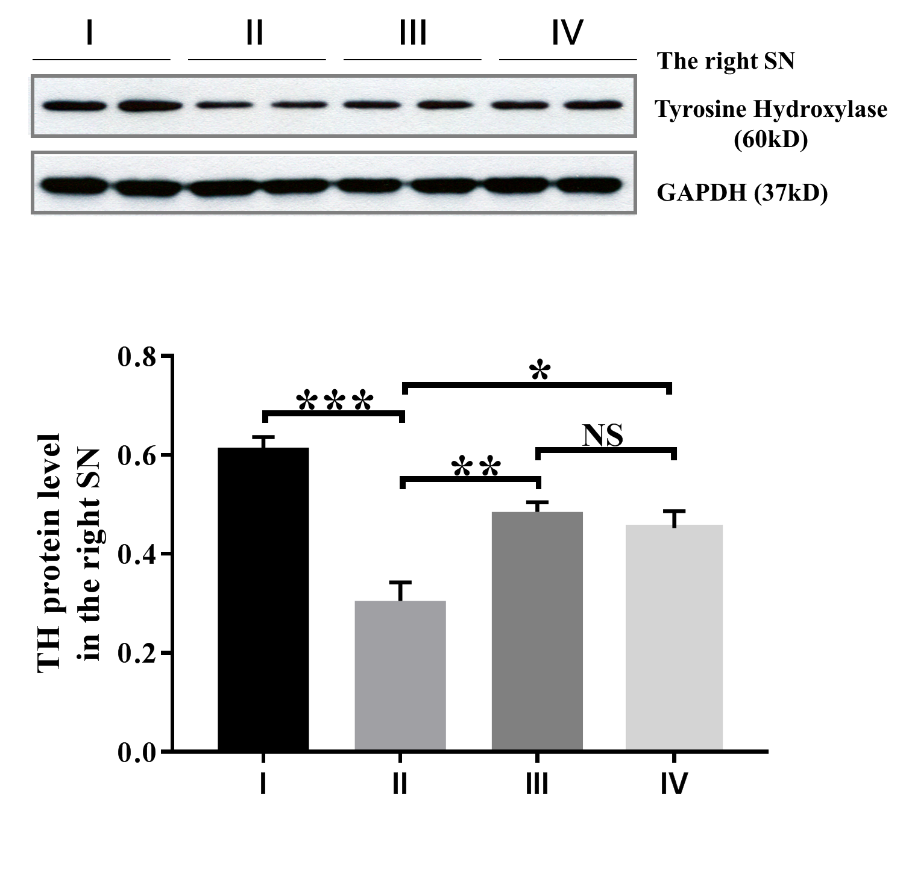


**Supplementary Figure 2** Neuroprotective effects of STN-UDBS or GP-UDBS in MPTP mice. Western blot analysis of TH protein level indicated that TH protein level in the right SN was decreased after MPTP injection, and UDBS restored this situation. (group I: control-sham, group II: MPTP-sham, group III: MPTP-STN-UDBS and group IV: MPTP-GP-UDBS; one–way ANOVA with Tukey’s post hoc: *p < 0.05, **p < 0.01, ***p < 0.001, mean ± SEM, n = 4 per group)


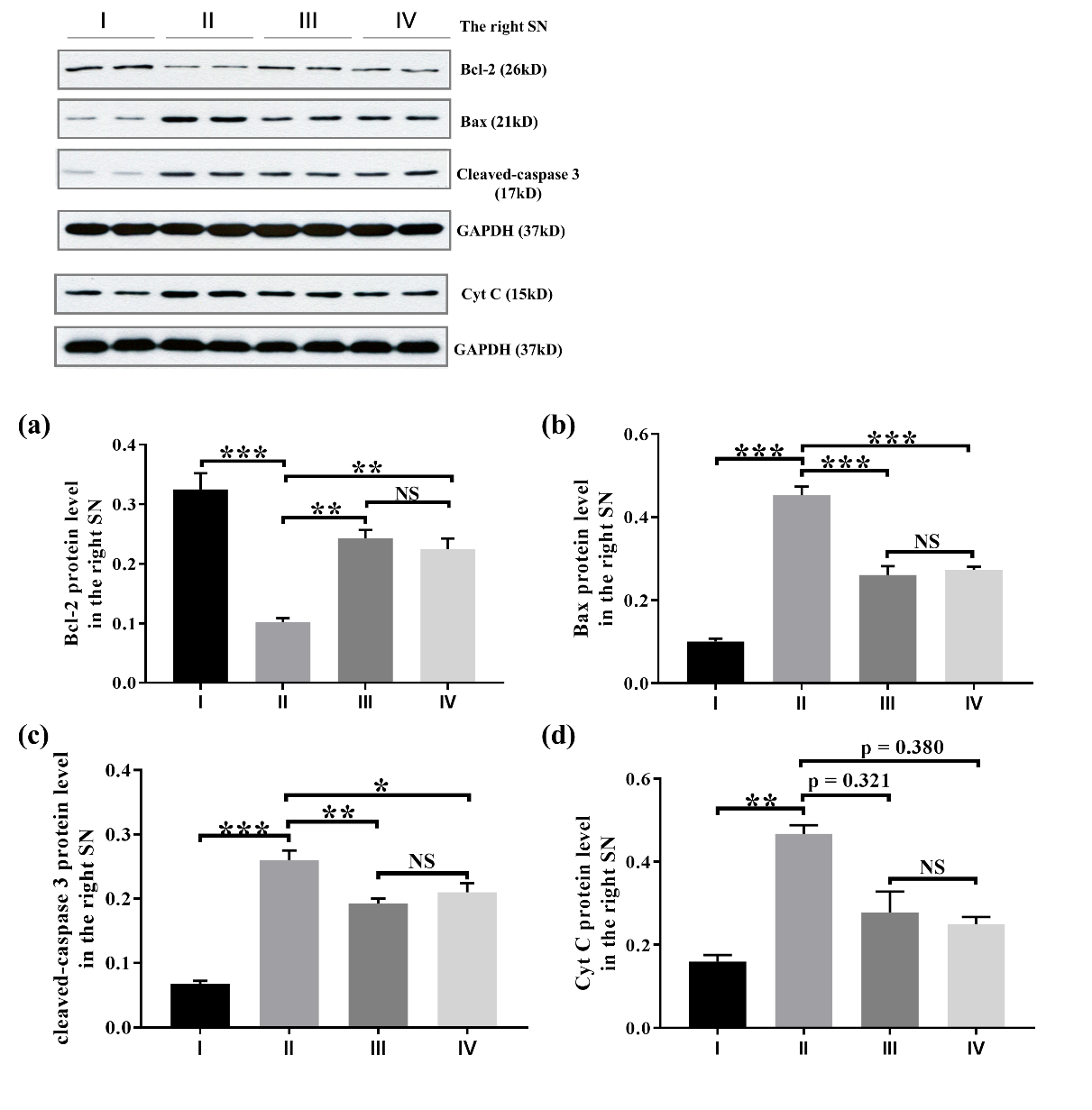


**Supplementary Figure 3** UDBS suppressed MPTP induced apoptosis in the right SN. Bcl-2 (a) was decreased and Bax (b) was increased after MPTP injection, which were restored by ultrasound stimulation. Besides, UDBS downregulated cleaved-caspase 3 (c) and Cyt C level (d) which were increased by MPTP injection. (group I: control-sham, group II: MPTP-sham, group III: MPTP-STN-UDBS and group IV: MPTP-GP-UDBS; one–way ANOVA with Tukey’s post hoc: *p < 0.05, **p < 0.01, ***p < 0.001 for Bcl-2, Bax and cleaved-caspase 3 analysis; Kruskal Wallis nonparametric ANOVA for Cyt C analysis; mean ± SEM, n = 4 per group).


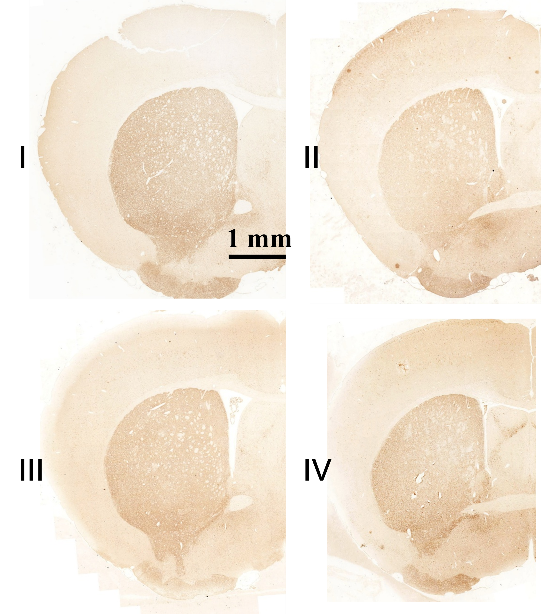


**Supplementary Figure 4** TH expression in the left striatum. Optical density of TH neurities was decreased by MPTP treatment, and UDBS increased optical density of TH neurities in striatum (group I: control-sham, group II: MPTP-sham, group III: MPTP-STN-UDBS and group IV: MPTP-GP-UDBS).


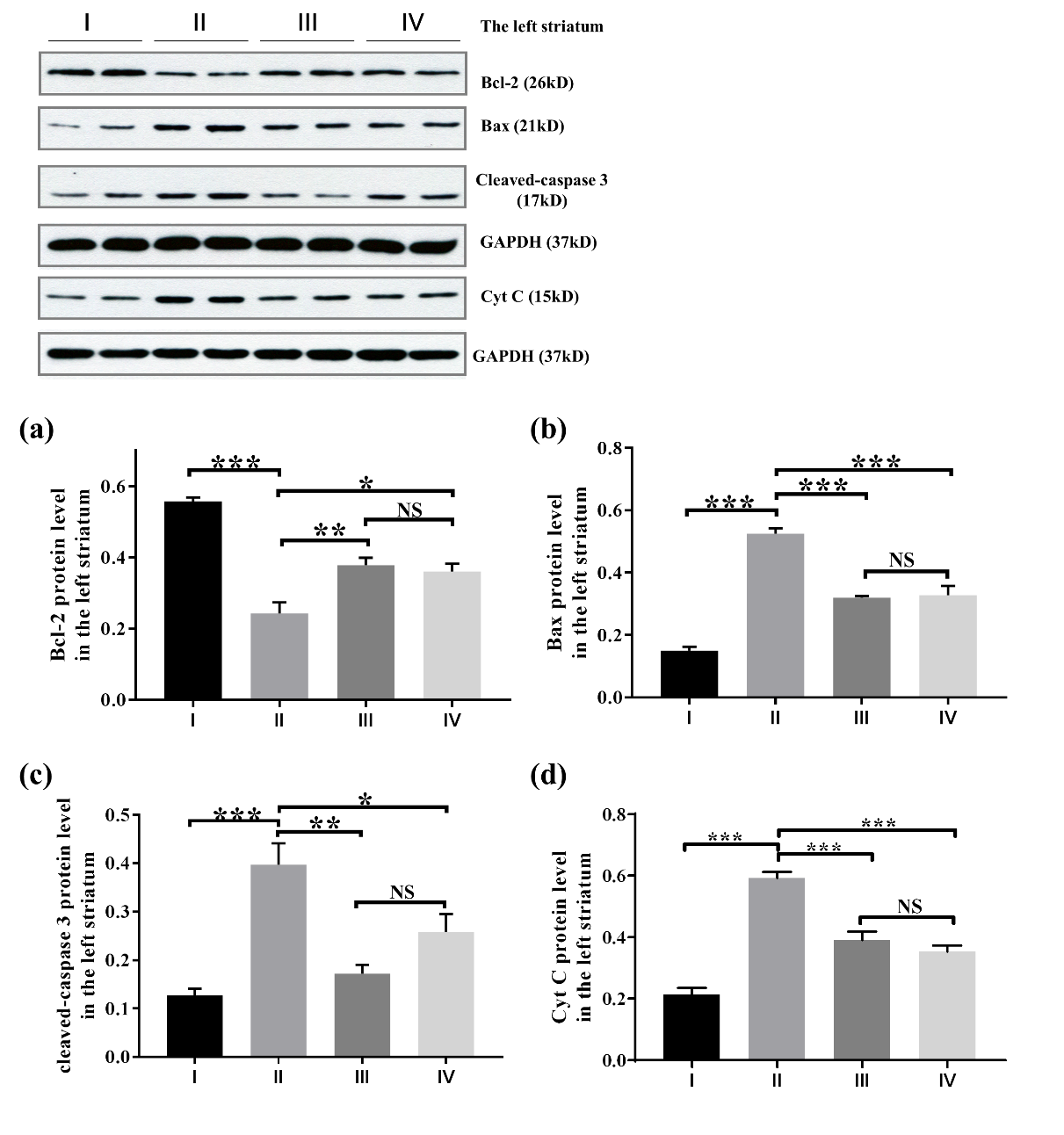


**Supplementary Figure 5** STN-UDBS and GP-UDBS suppressed MPTP induced cell apoptosis in the left striatum. Bcl-2 (a) was decreased and Bax (b) was increased in left striatum after MPTP administration, and this situation was reversed by ultrasound stimulation. UDBS decreased cleaved-caspase 3 (c) and Cyt C (d) activity which were enhanced after MPTP treatment (group I: control-sham, group II: MPTP-sham, group III: MPTP- STN-UDBS and group IV: MPTP-GP-UDBS; one–way ANOVA with Tukey’s post hoc: *p < 0.05, **p < 0.01, ***p < 0.001, mean ± SEM, n = 4 per group).


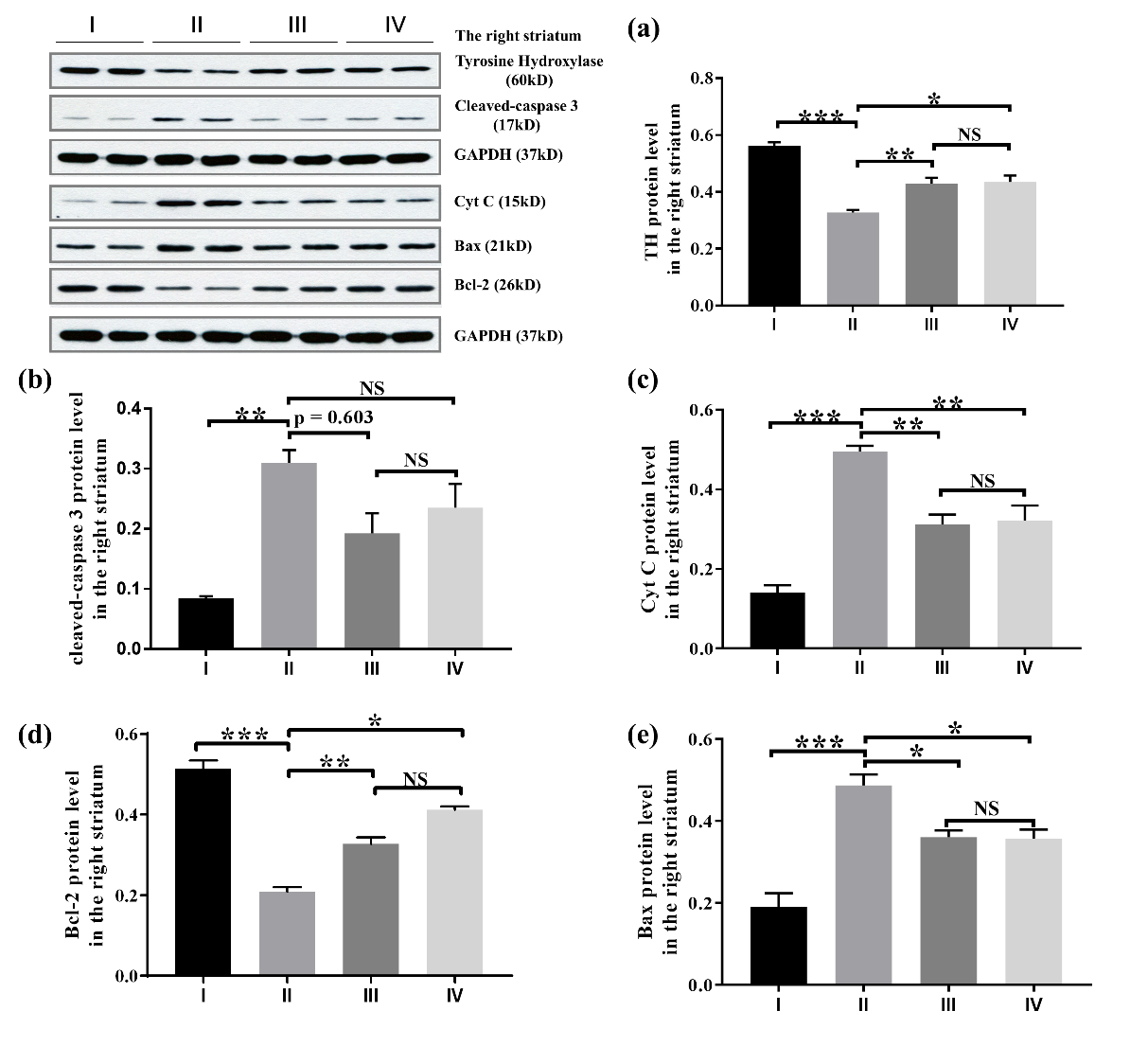


**Supplementary Figure 6** UDBS has neuroprotective effect in the right striatum. (a) Western blot analysis of TH protein level in the right striatum indicated that TH protein level was decreased after MPTP injection, however, UDBS increased TH protein level. UDBS restored cleaved-caspase 3 (b) and Cyt C (c) activity. Bcl-2 (d) was decreased and Bax (e) was increased after MPTP treatment, which were repaired by ultrasound stimulation. (group I: control-sham, group II: MPTP-sham, group III: MPTP-STN-UDBS and group IV: MPTP-GP-UDBS; one–way ANOVA with Tukey’s post hoc: *p < 0.05, **p < 0.01, ***p < 0.001 for TH, Bcl-2, Bax and Cyt C analysis; Kruskal Wallis nonparametric ANOVA for cleaved-caspase 3 analysis; mean ± SEM, n = 4 per group).

**Supplementary Figure 7** UDBS increased T-SOD in the left striatum. Striatal T-SOD in group II decreased compared with group I (group I: 1.00 ± 0.07, group II: 0.75 ± 0.04, p = 0.059) and increased in group III and group IV compared with group II (group III: 0.95 ± 0.07, p = 0.180; group IV: 0.84 ± 0.08, p = 0.477). There was no significant difference between group III and IV. (group I: control-sham, group II: MPTP-sham, group III: MPTP-STN-UDBS and group IV: MPTP-GP-UDBS; one-way ANOVA with Tukey’s post hoc, mean ± SEM, n=8 for each group).


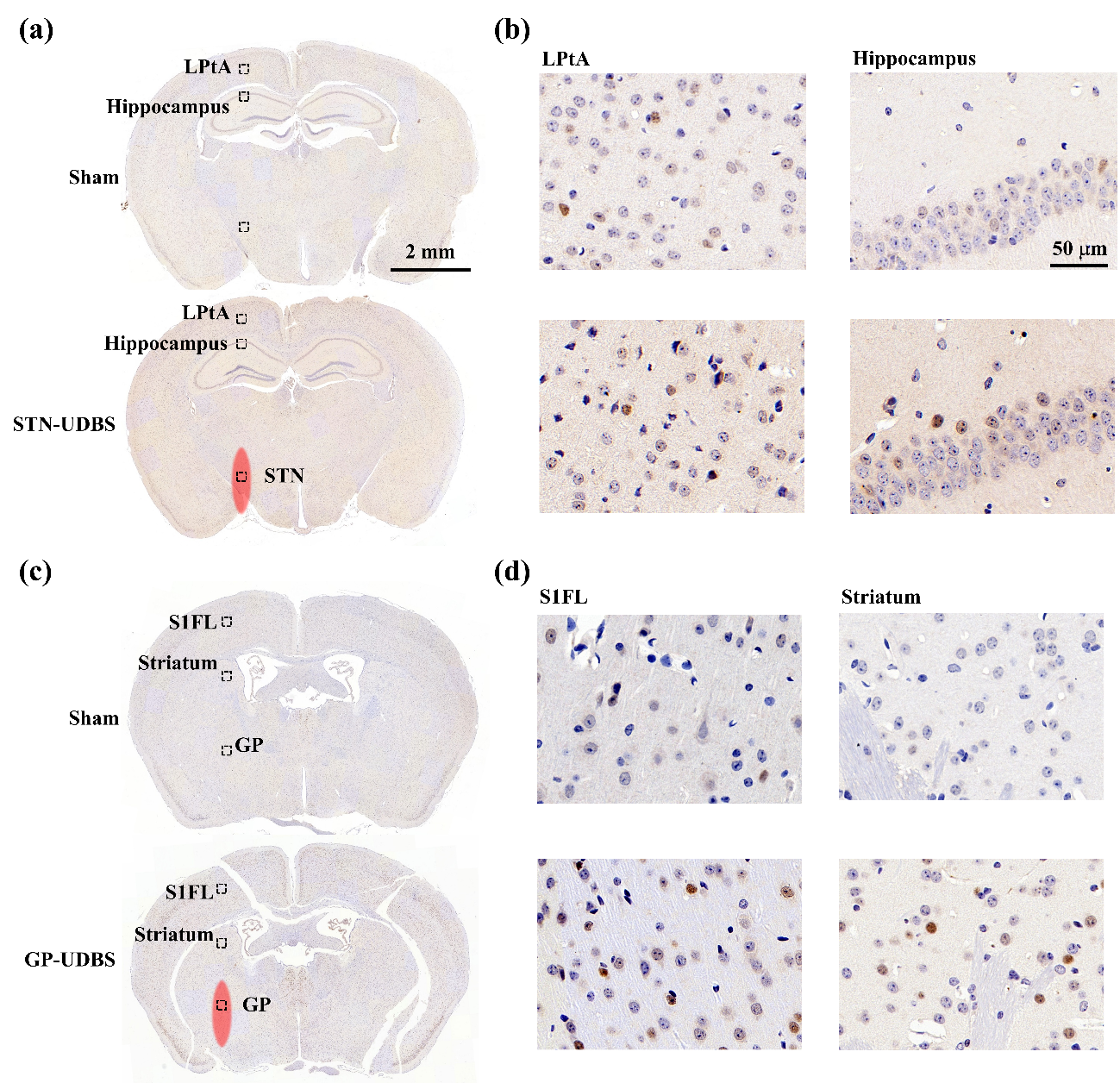


**Supplementary Figure 8** C-Fos expression in the route of ultrasound stimulation. (a, b) c-Fos expression in lateral parietal association cortex (PLtA) and Hippocampus. (c, d) C-Fos expression in primary somatosensory cortex, forelimb region (S1FL) and striatum. Cells with nuclear c-Fos staining (brown cell nuclei) represented cells that respond to ultrasound stimulation, and UDBS targeted regions were indicated by red ellipses.


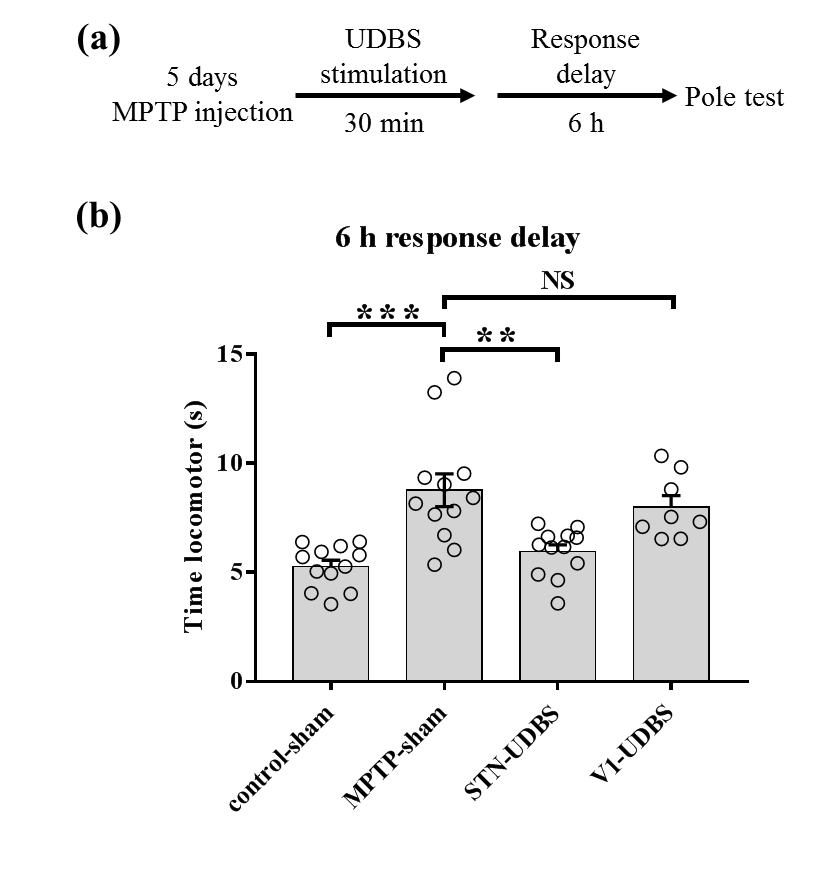


**Supplementary Figure 9** The effect of STN-UDBS on motor function in the pole test at 6 hours after ultrasound stimulation. (a) Time point for the pole test. (b) STN-UDBS improved motor behavior in MPTP mice at 6 hours after ultrasound stimulation, while ultrasound stimulation of primary visual cortex (V1) had little impact on behavior performance of MPTP mice. (One–way ANOVA with Bonferroni’s post hot: *p < 0.05, **p < 0.01, ***p < 0.001, mean ± SEM, n = 12 for control-sham, MPTP-sham and STN-UDBS group, n = 8 for V1-UDBS group).


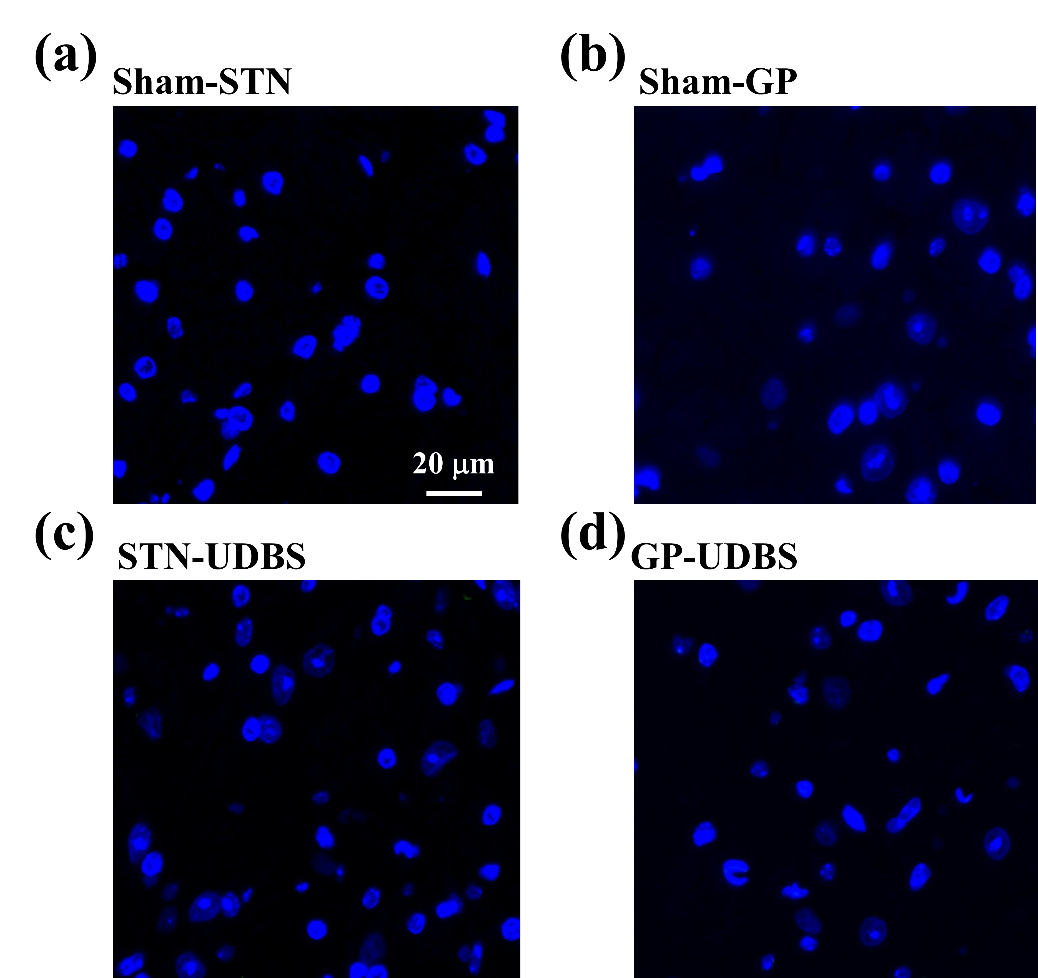


**Supplementary Figure 10** TUNEL staining in STN and GP after ultrasound stimulation indicated that no apoptosis was induced by ultrasound stimulation.
